# Narcissism, national narcissism, COVID-19 conspiracy belief, and social media use as predictors of compliance with COVID-19 public health guidelines

**DOI:** 10.1007/s12144-022-03715-6

**Published:** 2022-10-05

**Authors:** Stein Vaal, Malcolm B. Schofield, Ian S. Baker, Ben L.H. Roberts

**Affiliations:** University of Derby, Kedleston Road, DE22 1GB Derby, United Kingdom

**Keywords:** COVID-19, Conspiracy theory, Narcissism, National narcissism, Social media use, COVID-19 public health guidelines, Health Belief Model

## Abstract

Understanding health belief models, and the variables that influence adherence to public health measures imposed by local governments and international health bodies, is crucial to slowing down the spread of the virus that causes COVID-19. Conspiracy theories about the virus have quickly spread on social media and have been linked to reluctance to comply with COVID-19 regulations. Personality traits such as narcissism and collective national narcissism have also been associated with the way we perceive severity and susceptibility to the disease. To examine this further, participants (N = 183) completed an online questionnaire measuring belief in COVID-19 conspiracies, trait narcissism, national narcissism, and social media usage. A model containing these variables was able to significantly predict adherence to COVID-19 preventative health behaviours, with higher levels of COVID-19 conspiracy belief, narcissism, and social media usage all contributing to reduced adherence to recommended COVID-19 health behaviours. The findings suggest conspiracy beliefs, narcissism, and social media play a key role in adherence to behaviours orientated towards stopping the spread of COVID-19. Governments and social media companies need to demonstrate greater awareness of the negative effects of conspiracy theories spread through social media, in addition to awareness of how these effects may be greater in more narcissistic individuals.

## Introduction

According to the Health Belief Model (HBM; Janz & Becker, 1984), individuals’ perception of the threat and severity of global health emergencies is likely to impact their motivation to adhere to health behaviours. Variables that impact such health beliefs, including personality traits, would therefore be likely to influence whether individuals comply with government-imposed preventative health measures, such as those instigated due to the coronavirus disease 2019 (COVID-19) pandemic. COVID-19 is an infectious respiratory disease caused by severe acute respiratory syndrome coronavirus 2 (SARS-CoV-2). It was first identified in Wuhan, China, in December 2019, but quickly spread around the world; on January 20th, 2020, the World Health Organization (WHO) declared it as a global health emergency (WHO, 2021). Subsequently, many governments imposed substantial public health measures designed to limit the spread of the virus, including “lockdowns”, social distancing, and work-from-home orders (Haug et al., 2020).

Previous research has indicated a possible relationship between COVID-19 health behaviour and the personality trait of narcissism, which is characterized by an inflated, grandiose self-image, a need for continual external recognition, and low regard for others (Morf & Rhodewalt, 2001). Key aspects of narcissism, such as entitlement and greater self-interest, have been found to be associated with lower compliance with government public health guidelines, with those higher in these characteristics less likely to socially distance and adhere to government-imposed preventative health behaviours related to COVID-19 (Oosterhoff & Palmer, 2020; Zitek & Schlund, 2021). Due to the self-centred nature of narcissism, those high in this trait may be less likely to adopt prosocial health behaviours such as mask-wearing and social distancing, as they see less immediate personal gain from such behaviours and are less concerned with how their behaviour might affect others. Narcissistic individuals may also see government-imposed COVID-19 regulations as an attack on their autonomy and thus shift into antisocial tendencies in order to maintain their sense of superiority and control. Alternatively, narcissists who rely heavily on others for admiration may adhere to government guidelines in order to receive praise and recognition from those around them. In support of the former, several recent studies have found negative relationships between narcissism and the adoption of public and private hygiene behaviours and regulations tailored towards minimising the spread of the virus (Nowak et al., 2020; Otterbring et al., 2021; Triberti et al., 2021).

The relationship between narcissism and COVID-19 preventive health behaviours maybe related to health beliefs. The HBM suggests reduced motivation to engage in preventative health behaviours is driven by health beliefs such as lower perceived susceptibility to disease or infection (Janz & Becker, 1984), a relationship which has been replicated with respect to the current pandemic (Dryhurst et al., 2020). Although higher levels of trait narcissism have been found to predict lower perceived susceptibility to COVID-19, there have been mixed findings on the relationships between sub-types of narcissism and perceived susceptibility. When differentiating between two different dimensions of narcissism identified by Back et al. (2013), namely narcissistic admiration and narcissistic rivalry, studies found narcissistic admiration predicted greater – rather than lower – perceived susceptibility to COVID-19 (Nowak et al., 2020), whereas rivalrous narcissism appears to predict lower perceived susceptibility to infection in some studies but not others (Zajenkowski et al., 2020; Venema & Pfattheicher, 2021).

Considering wider group dynamics, collective narcissism extends the concept of narcissism into the intergroup domain and is defined as an unrealistic exaggerated belief in the greatness of one’s in-group and grievance for lack of external appreciation and recognition (Golec de Zavala & Lantos, 2020; Żemojtel-Piotrowska et al., 2021). Individuals high in collective narcissism are focused on maintaining a positive image of their in-group; due to this preoccupation, they exhibit hypersensitivity to in-group threat (Golec de Zavala, 2018). This hypersensitivity leads them to foster convictions that others are acting malevolently against their in-group. This relationship works to protect the individuals’ positive image of the group, as any failings by the in-group can be attributed to secret malevolent acts of out-group members, rather than the group’s own shortcomings. Van Bavel et al. (2020) found national narcissism (collective narcissism measured with respect to one’s nationality) to be associated with greater support for COVID-19 public health measures as well as greater self-reported physical hygiene. However, there have been mixed findings from other recent studies, which have found national narcissism to be either unrelated to prevention, or only related to certain preventative behaviours such as handwashing (Sternisko et al., 2020; Nowak et al., 2020). One possible explanation for these inconsistencies is that collective narcissists have been shown to prioritise their own needs, and the protection of the in-group image, over the well-being of fellow citizens (Cichocka, 2016; Marchlewska et al., 2020). Therefore, in some cases national narcissists may adhere to preventative health behaviours that protect themselves (e.g., handwashing) but may not adhere to behaviours designed to protect others (e.g., wearing a facemask). This relates to the “perceived benefits” dimension of the HBM (Janz & Becker, 1984), with individuals more likely to act where they perceive a greater benefit to their actions.

The conviction that others are acting malevolently against the in-group may lead to belief in conspiracy theories. Conspiracy theories are alternative explanations for significant social or political events, usually assuming secret or malevolent plots by two or more powerful actors (Douglas et al., 2019). Research suggests individuals may believe in conspiracy theories to satisfy important social psychological desires for understanding, certainty, control, security, and maintenance of a positive image of the self or the group (Douglas et al., 2017). Although some consequences of conspiracy theories can be positive (e.g., the revelation of inconsistencies in official versions of events or raising issues in society that need to be addressed), the majority of consequences appear to manifest in negative ways (e.g., prejudice, violence, and other antisocial behaviour). Previous research has identified a strong association between medical conspiracy beliefs and health choices, such as engaging with medical professionals, trusting alternative medical advice sources, and choosing unconventional medicines (Oliver & Wood, 2014). Conspiracy belief is very likely to increase during crises such as a global health pandemic, as these events generate aversive feelings of uncertainty and lack of control which stimulate a need to make sense of the situation, possibly through belief in a conspiracy related to the event. It has been noted that, throughout history, social crises have correlated with an increase in conspiratorial thinking (van Prooijen & Douglas, 2017). Due to the magnitude of these events individuals may seek out an explanation of similar magnitude; conspiracy theories provide such an explanation as they involve secret malevolent plots which parallel in perceived importance to the social or political event or circumstance (Leman & Cinnerella, 2007).

Recent research into the possible effects of conspiracy beliefs on the current COVID-19 pandemic have generated concerning results regarding people’s intentions to comply with government recommendations. In relation to the HBM, a belief that the threat from COVID-19 is either fabricated or exaggerated would be expected to lead to lower perceived severity and susceptibility, and therefore a lower likelihood of action. Indeed, several studies have found belief in COVID-19 conspiracy theories to be negatively associated with preventative intentions and actions (Allington & Dhavan, 2020; Allington et al., 2020; Barua et al., 2020; Freeman et al., 2020; Romer & Jamieson, 2020). One explanation for these findings is that need for uniqueness (a factor previously linked to conspiracy mentality) may make individuals more likely to adopt atypical behaviours (Lynn & Snyder, 2002) and not comply with descriptive social norms. This need for uniqueness could be linked to several personality traits, although narcissism would appear to be of particular importance.

Narcissism has been repeatedly linked with social media usage, and social media platforms have been identified as the main vectors for dissemination of COVID-19 conspiracy theories (AVAAZ, 2020). Considering individuals high in narcissism are excessively preoccupied with how people view them, it appears logical for them to utilise these media platforms which allow them to broadcast themselves in self-enhancing ways to a previously unreachable audience, ostensibly appealing to their desires for attention and praise (Buffardi & Campbell, 2008). McCain and Campbell (2018) found narcissism was related to more time spent on social media, as well as more posts and more friends/followers, a finding which was replicated by Singh et al. (2018). As narcissism and conspiracy theories are related to adherence to COVID-19 preventative behaviours, social media usage may also play a role in hindering compliance with these public health behaviours. Supporting this, Allington et al. (2020) found a negative association between the use of social media as a source of information about COVID-19 and preventative health behaviours, suggesting that the higher an individual’s social media usage, the lower their likelihood of engaging with health behaviours aimed at preventing the spread of COVID-19.

While previous research has therefore determined some important relationships between these variables, they have not yet been examined together in a single study, and some inconsistencies have been found. The present study therefore aimed to address these issues by examining COVID-19 conspiracy theory beliefs, social media use, narcissism and national narcissism to create a model predicting adherence to COVID-19 health behaviours. In line with previous research, the hypothesis for the study was that lower levels of COVID-19 conspiracy belief, lower levels of narcissism, higher levels of collective narcissism, and lower levels of social media use would significantly predict higher levels of compliance with COVID-19 preventative health behaviours.

## Method

### Design

A correlational design was used. COVID-19 conspiracy belief, social media usage, and the traits of narcissism and collective narcissism operated as predictor variables for the outcome variable – the extent of compliance by UK residents with COVID-19 public health regulations imposed by the UK government. All data was collected in line with the British Psychological Society’s (BPS) ethical guidelines, with ethical clearance for this study being granted by the University of Derby’s Psychology ethics committee (ref number ETH2021-1104).

### Participants

After removing 32 incomplete responses (85.1% completion rate), participants (N = 183) consisted of 143 (78.1%) females (mean age = 35.12, SD = 13.8), 37 (20.2%) males (mean age = 43.16, SD = 15.04), and 3 (1.6%) non-binary/other/preferred not to say. Most participants (163, 89.1%) identified as British, with 16 nationals from other European countries, 3 Americans and 1 Chinese. They were an opportunity sample recruited through online social media, online adverts, word-of-mouth, and the University of Derby’s internal Psychology Research Participation Scheme. The exclusion criteria were that individuals could not be under the age of 18 and could not currently live outside the UK, as this study used UK-specific COVID-19 public health regulations.

### Materials

The survey was administered online using the Qualtrics platform, and consisted of the following scales:

#### Narcissistic Personality Inventory-16 (NPI-16)

The Narcissistic Personality Inventory-16 (NPI-16; Ames et al., 2006) consists of 16 forced-choice pairs of statements such as “I really like to be the centre of attention” and “It makes me uncomfortable to be the centre of attention”; individuals must choose which statement is most self-descriptive. Narcissistic statements score 1, while non-narcissistic statements score 0. The scale is scored out of 16, with higher scores indicating higher levels of narcissism. The scale shows notable convergent, discriminant and predictive validity (Ames at al., 2006; Gentile et al., 2013). The Cronbach’s alpha for this study was 0.72 (Ames et al., 2016), indicating a good level of internal reliability.

#### Collective Narcissism Scale (CNS)

The Collective Narcissism Scale (CNS; Golec de Zavala et al., 2009) is a 9-item scale measuring collective narcissism, an intergroup domain of narcissism centred around the unrealistic belief in the greatness of a particular group. Participants are instructed to think about a specific group while ranking items such as “It really makes me angry when others criticize my group” on a 6-point Likert scale from “I strongly disagree” (representing a score of 1) to “I strongly agree” (representing a score of 6). The scale is scored out of 54 with a higher score indicating higher collective narcissism. This scale showed good internal reliability in this study, with a Cronbach’s alpha of 0.86.

#### General Social Media Usage Subscale (GSMUS)

The General Social Media Usage Subscale (GSMUS) is a component of the Media and Technology Usage and Attitudes Scale devised by Rosen et al. (2013) and is used to measure the extent to which individuals use social media. The subscale consists of 9 frequency-based questions such as “Check social media from your smartphone”, which individuals must answer using a Likert scale from 1 (Never) to 10 (All the time). Higher scores on this scale indicate higher levels of social media usage. Although it makes up part of a larger scale, the GSMUS is internally reliable and externally valid when used independently (Rosen et al., 2013). The Cronbach’s alpha for this subscale in this sample was 0.83.

#### COVID-19 Conspiracy Belief Questionnaire

A short questionnaire was generated containing the most popular COVID-19 conspiracy theories circulating in December 2020 from various media sources, such as “COVID-19 was created artificially” and “COVID-19 is a hoax”. The 10-item questionnaire used a 5-point Likert scale (1= “strongly disagree”, 5= “strongly agree”) which individuals used to state their agreement with each conspiracy theory; this response format was used since previous research has been criticised for using imbalanced options that may have led to inflated agreement with conspiracy theories (Sutton & Douglas, 2020). The questionnaire was scored out of 50, with a high score indicating high COVID-19 conspiracy belief. This scale demonstrated good internal reliability (α = 0.87).

#### COVID-19 Preventative Health Behaviours Questionnaire

Compliance with COVID-19 public health recommendations imposed by the UK Government was measured via a 5-item questionnaire. Participants were asked to indicate how often they had been complying with the COVID-19 public health behaviours on a 5-point frequency-based Likert scale from 1 (Never) to 5 (Always). The items for the scale were obtained from the COVID-19 section of the UK Government website (GOV.UK, 2020). Examples of items on the scale include “Staying 2 metres apart from people you do not live with where possible, or 1 metre with extra precautions in place” and “Staying at home, only leaving your home where necessary”. This questionnaire had acceptable internal reliability in this sample, producing a Cronbach’s alpha of 0.74.

### Procedure

Once the participant accessed the survey, they were presented with information detailing the purpose of the study, what they would be required to do, their right to withdraw, and how their data would be stored and used. After the participant had consented to participate, demographic information was obtained (age, gender, nationality) and they were instructed to generate a unique code which they could use to withdraw their data from the study. The individuals were then presented with the three scales (NPI-16, CNS and GSMUS) and the two COVID-19 related questionnaires randomly to control for order effects. Once all scales and questionnaires were completed the participants were presented with debriefing information which outlined the aim of the study, what was being measured, and their right to withdraw their data. Participants were then thanked for their participation.

## Results

Statistical analysis of the data was performed using SPSS version 27. The descriptive statistics for COVID-19 conspiracy belief, narcissism, national narcissism, social media usage and COVID-19 preventative health behaviour are shown in Table 1.

Scatterplots were created between each predictor variable and the outcome variable to identify any outliers that appeared to not fit the general trend of the data. Cook’s Distance had a maximum value of  0.119, indicating these outliers were not problematic. The data produced a Durbin-Watson score of 2.117, indicating that the adjacent residuals appear not to be correlated. Correlations between the variables are shown in Table 1; VIF scores ranged between 1.076 and 1.135 suggesting no multicollinearity.

**Table 1:** Correlation coefficients (significance levels), means and SDs for the predictor and outcome variables

	COVID-19 Preventative Health Behaviour	COVID-19 Conspiracy Belief	Narcissism	National Narcissism	General Social Media Use
COVID-19 Conspiracy Belief	− 0.412**				
Narcissism	− 0.223**	0.152*			
National Narcissism	− 0.182*	0.251**	0.273**		
General Social Media Use	− 0.191**	0.126*	0.100	− 0.022	
Mean	20.82	19.56	2.36	22.96	47.49
SD	3.71	7.85	2.50	8.40	11.77

** Correlation is significant at the 0.01 level (2-tailed). * Correlation is significant at the 0.05 level (2-tailed)

Data were analysed using a multiple regression using the forced entry method. The regression equation produced a medium-to-large effect size (*R*^*2*^ = 0.215, *R*^*2*^_*adj*_ = 0.197) indicating that, together, COVID-19 conspiracy belief, narcissism, national narcissism, and social media use significantly predicted compliance with COVID-19 preventative health behaviours, *F* (4,178) = 12.170, *p* < .001.

There was a significant negative relationship between COVID-19 conspiracy belief and COVID-19 preventative health behaviours (β = − 0.360, p < .001), a significant negative relationship between narcissism and COVID-19 preventative health behaviours (β = − 0.139, p = .047), and a significant negative relationship between social media use and COVID-19 preventative health behaviours, (β = − 0.133, p = .050). However, the relationship between national narcissism and COVID-19 preventative health behaviours was non-significant, (β = − 0.056, p = .428).

## Discussion

### Findings

The main finding of this study demonstrates a model consisting of measures of COVID-19 conspiracy belief, narcissism, national narcissism, and social media use was able to significantly predict 21.5% of the variance in how well individuals complied with preventative behaviours designed to combat the spread of COVID-19 in the UK. Individuals high in COVID-19 conspiracy belief, trait narcissism and social media use were found to be less likely to adhere to preventative health behaviours. No significant relationship was found between collective national narcissism and degree of compliance with COVID-19 health behaviours.

Recent research conducted during the pandemic indicated that belief in conspiracy theories led to reduced compliance with public health measures designed to reduce the spread of COVID-19 (Haug et al., 2020). The findings from this research support the hypothesis and give credence to previous studies (Allington & Dhavan, 2020; Allington et al., 2020; Barua et al., 2020; Freeman et al., 2020; Romer & Jamieson, 2020), in accordance with those studies COVID-19 conspiracy belief was found to predict lower levels of compliance with public health measures employed by the UK government. As per the HBM (Janz & Becker, 1984), individuals who believe COVID-19 not to be a genuine threat will perceive low levels of severity and susceptibility, and are therefore less likely to act to reduce that threat. In order to address previous criticisms regarding imbalanced options potentially leading to inflated agreement with conspiracy theories (e.g., Sutton & Douglas, 2020) the present study provided a balanced response selection so participants could both equally agree and disagree with each conspiracy theory. As the present study’s results are consistent with those of previous research, it suggests that although previous studies may have exaggerated the prevalence of COVID-19 conspiracy theory belief, this inaccuracy did not appear to affect the overall relationship between conspiracy belief and compliance with preventative health behaviours.

The self-obsessed nature of narcissism, coupled with low regard for others, explains the finding that narcissism was negatively associated with COVID-19 preventative health behaviour adherence. The findings of this research support the hypothesis, which was based on previous findings that higher levels of trait narcissism were related to health behaviour during the pandemic (e.g., Nowak et al., 2020). Although compliance with government-imposed public health behaviours may represent an opportunity for narcissists to receive praise from others, their prioritisation of personal needs, their sense of entitlement, and their need for control, seem to contribute more strongly to their lack of adherence to COVID-19 preventative health behaviours; furthermore, narcissistic individuals may perceive themselves as less susceptible to infection due to overconfidence and a high sense of control (e.g. Venema & Pfattheicher, 2021). Collective narcissism, however, was not a significant predictor of adherence to recommended COVID-19 health behaviours. While this did not support the hypothesis, it was in line with the mixed findings of previous research and suggests that the national narcissist may only focus on their own needs, and the protection of the in-group image, rather than the wellbeing of other people (Cichocka, 2016; Marchlewska et al., 2020). Therefore, considering the “perceived benefits” dimension of the HBM, it is plausible that collective narcissism has no bearing on adherence to recommended COVID-19 health behaviours due to it concerning other people, whereas narcissism does predict adherence to recommended COVID-19 health behaviour due to it being solely based on the individual.

Social media usage was hypothesized to be negatively related to COVID-19 preventative health behaviours, due to the prevalence of conspiracy theories and misinformation on social media which negatively impact health beliefs about the pandemic. The findings from this study support this hypothesis and provide further evidence for a link between social media usage and non-engagement with these public health guidelines. Social media use and conspiracy belief shared a significant positive, albeit weak, correlation; it could be suggested from this finding that the degree of social media use may play a role in the accessibility of pandemic-related conspiracy theories, and hence their influence on the general population, as hypothesized by Allington et al. (2020).

### Implications, limitations and suggestions

This study has made an important contribution to the understanding of individuals’ compliance with government-imposed preventative health measures. The study’s findings demonstrate clearly the importance of tackling the proliferation of misinformation and conspiracy theories, particularly on social media, to ensure that individuals access accurate and useful health information. The finding of a link with narcissism suggests that, as recommended by Venema and Pfattheicher (2021), particular attention should be paid to designing campaigns to target groups known to be high in narcissism, such as men and young adults.

The study was limited to participants currently living in the UK – a more individualistic culture – so future research could attempt to replicate this study with participants living in more collectivist cultures, which may possess a greater collective identity and thus possibly demonstrate greater levels of collective national narcissism. Alternatively, countries who show high levels of patriotism and nationalism may also exhibit greater levels of national narcissism, which could lead to a stronger relationship between national narcissism and preventative health behaviours. Furthermore, more in in-depth measures of social media use would be helpful. In addition, a closer examination of different dimensions of narcissism, such as narcissistic admiration and narcissistic rivalry (Back et al., 2013), will allow a more nuanced understanding of the relationship between narcissism and these behaviours.

### Conclusion

In conclusion, this study suggests COVID-19 conspiracy belief, narcissism, and social media use can negatively influence individuals’ adherence to health behaviours recommended by the UK to combat the spread of the virus. The impact of social media during a public health crisis cannot be ignored; the vast amount of misinformation and conspiracy theories present on social media makes it a breeding ground for inaccurate information, in a period where people are using these platforms to actively search for knowledge and explanations for a novel and threatening scenario. As such, research should seek to find ways to combat the spread of misinformation and use the power of social media to deliver successful public health campaigns to the general population.

## Data Availability

The datasets generated during and/or analysed during the current study are available from the corresponding author on reasonable request.
